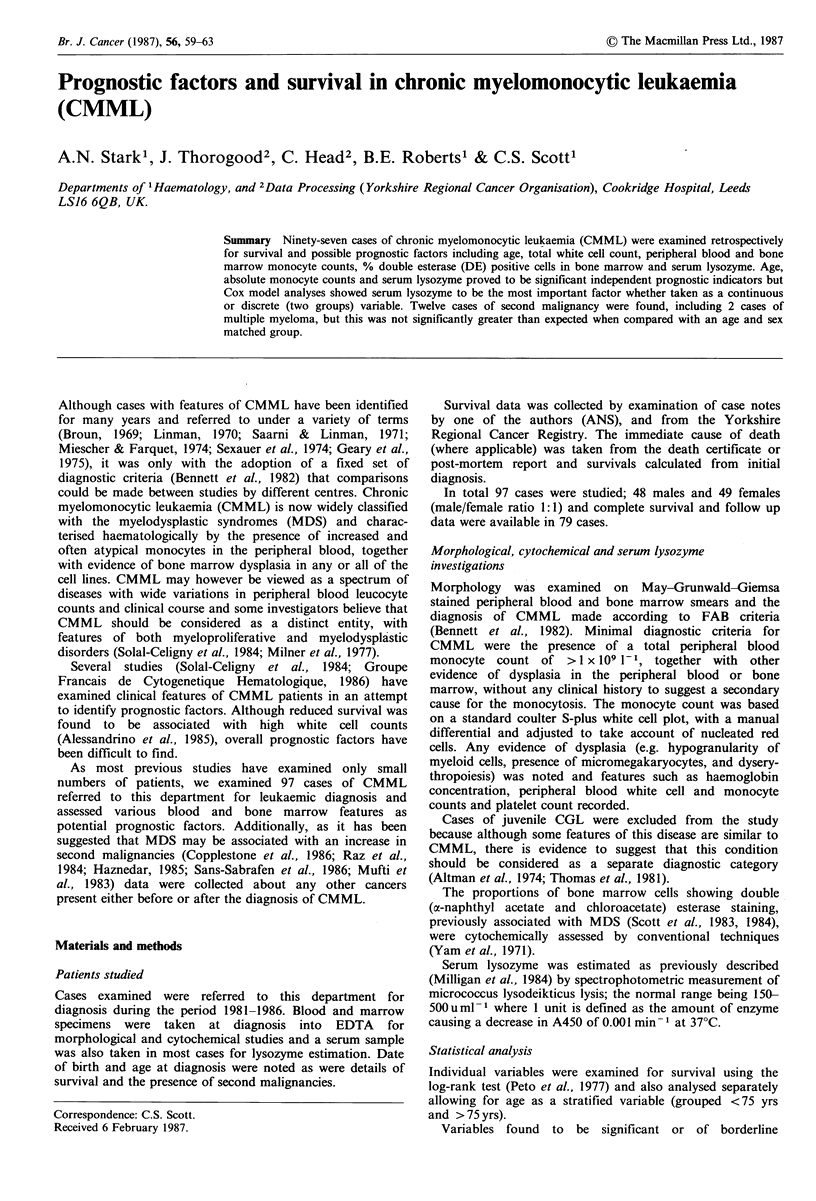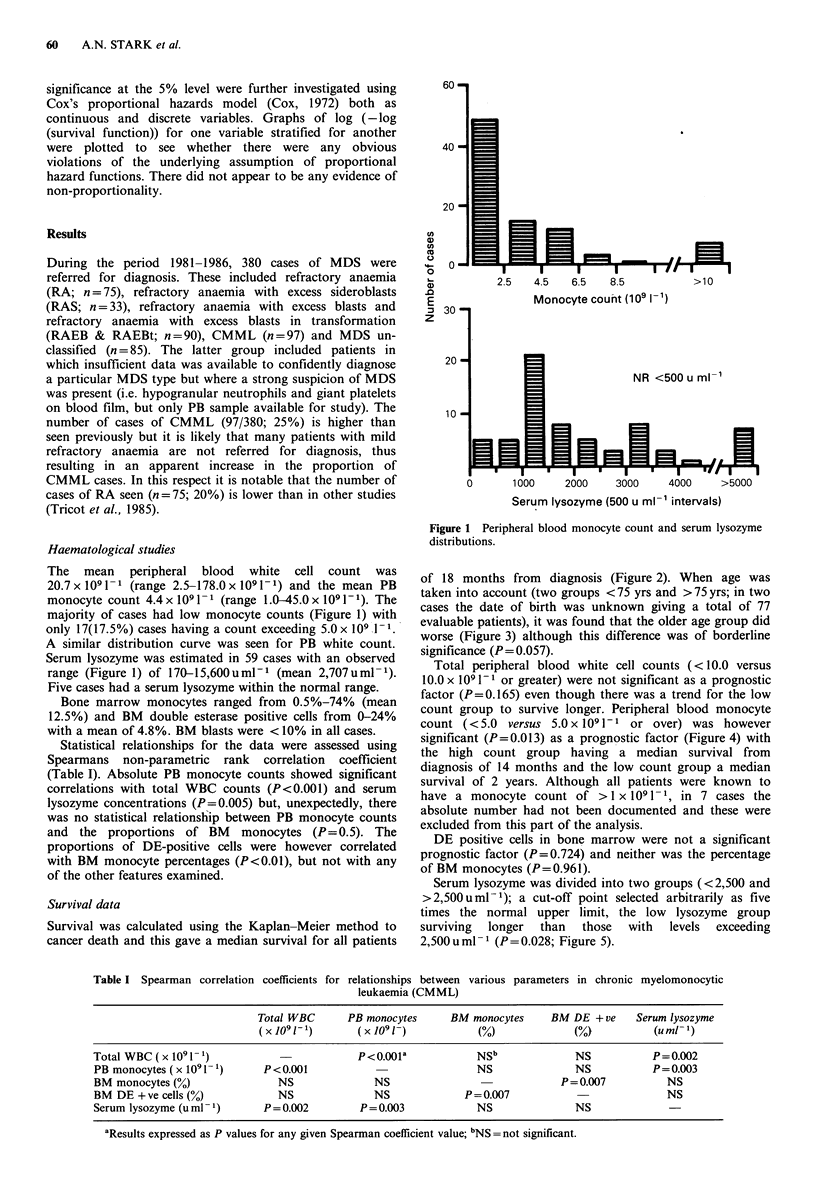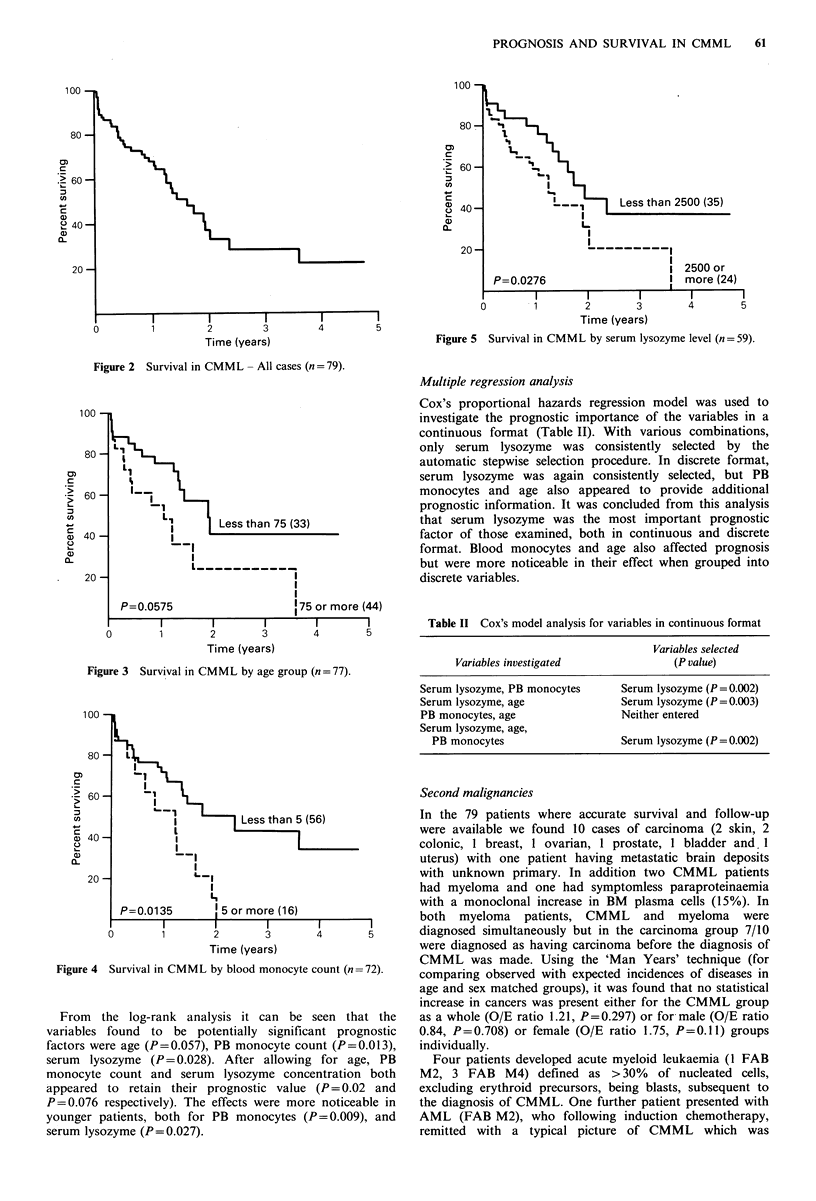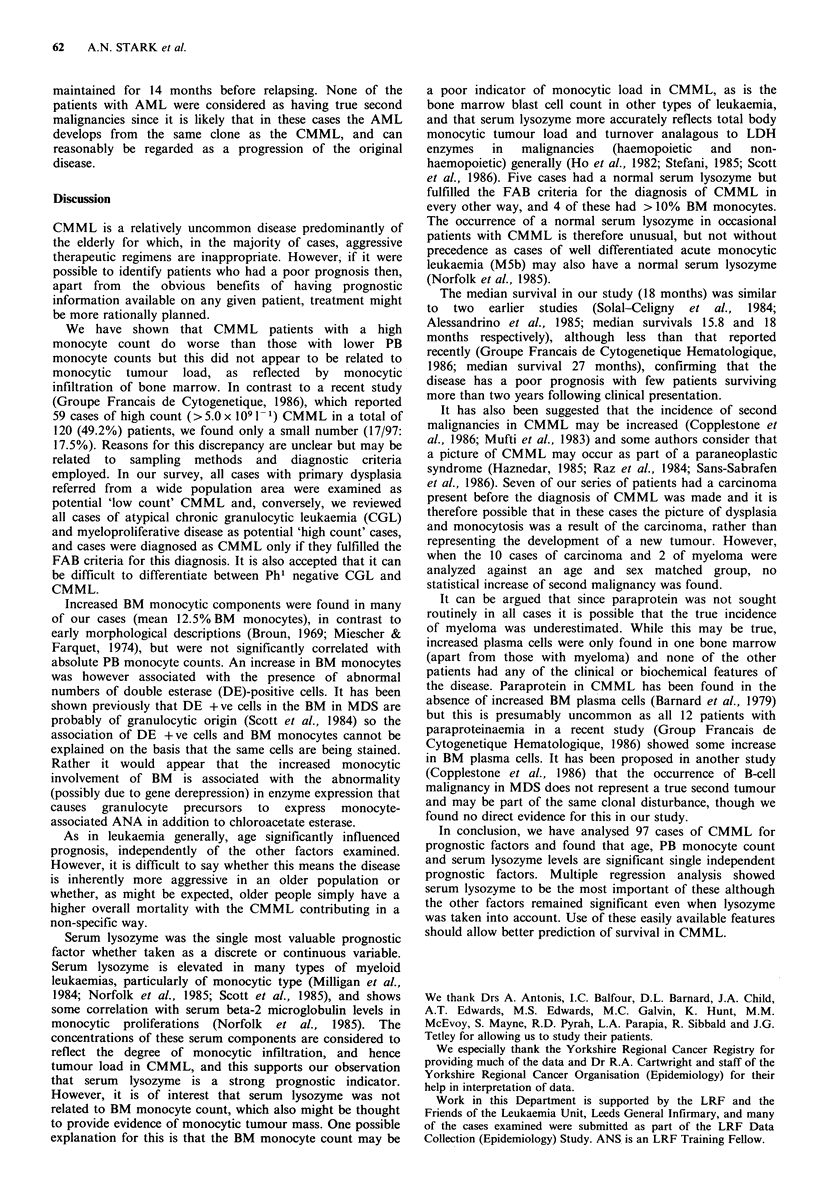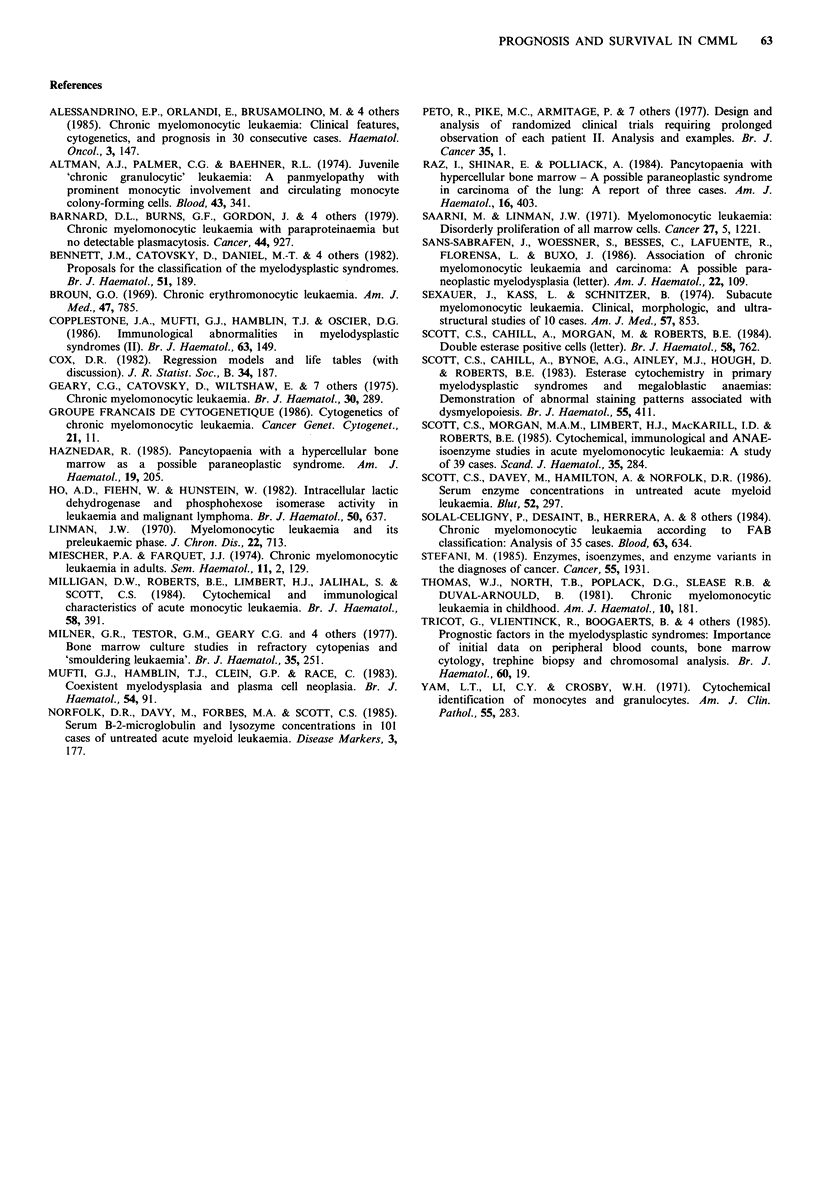# Prognostic factors and survival in chronic myelomonocytic leukaemia (CMML).

**DOI:** 10.1038/bjc.1987.154

**Published:** 1987-07

**Authors:** A. N. Stark, J. Thorogood, C. Head, B. E. Roberts, C. S. Scott

## Abstract

Ninety-seven cases of chronic myelomonocytic leukaemia (CMML) were examined retrospectively for survival and possible prognostic factors including age, total white cell count, peripheral blood and bone marrow monocyte counts, % double esterase (DE) positive cells in bone marrow and serum lysozyme. Age, absolute monocyte counts and serum lysozyme proved to be significant independent prognostic indicators but Cox model analyses showed serum lysozyme to be the most important factor whether taken as a continuous or discrete (two groups) variable. Twelve cases of second malignancy were found, including 2 cases of multiple myeloma, but this was not significantly greater than expected when compared with an age and sex matched group.


*Br. J. Cancer* (1987), **56**, 59–63 © The Macmillan Press Ltd., 1987

### Prognostic factors and survival in chronic myelomonocytic leukaemia (CMML)

A.N. Stark[1], J. Thorogood[2], C. Head[2], B.E. Roberts[1] & C.S. Scott[1]

*Departments of [1]Haematology, and [2]Data Processing (Yorkshire Regional Cancer Organisation), Cookridge Hospital, Leeds LS16 6QB, UK.*

**Summary** Ninety-seven cases of chronic myelomonocytic leukaemia (CMML) were examined retrospectively for survival and possible prognostic factors including age, total white cell count, peripheral blood and bone marrow monocyte counts, % double esterase (DE) positive cells in bone marrow and serum lysozyme. Age, absolute monocyte counts and serum lysozyme proved to be significant independent prognostic indicators but Cox model analyses showed serum lysozyme to be the most important factor whether taken as a continuous or discrete (two groups) variable. Twelve cases of second malignancy were found, including 2 cases of multiple myeloma, but this was not significantly greater than expected when compared with an age and sex matched group.

Although cases with features of CMML have been identified for many years and referred to under a variety of terms (Broun, 1969; Linman, 1970; Saarni & Linman, 1971; Miescher & Farquet, 1974; Sexauer *et al.*, 1974; Geary *et al.*, 1975), it was only with the adoption of a fixed set of diagnostic criteria (Bennett *et al.*, 1982) that comparisons could be made between studies by different centres. Chronic myelomonocytic leukaemia (CMML) is now widely classified with the myelodysplastic syndromes (MDS) and characterised haematologically by the presence of increased and often atypical monocytes in the peripheral blood, together with evidence of bone marrow dysplasia in any or all of the cell lines. CMML may however be viewed as a spectrum of diseases with wide variations in peripheral blood leucocyte counts and clinical course and some investigators believe that CMML should be considered as a distinct entity, with features of both myeloproliferative and myelodysplastic disorders (Solal-Celigny *et al.*, 1984; Milner *et al.*, 1977).

Several studies (Solal-Celigny *et al.*, 1984; Groupe Francais de Cytogenetique Hematologique, 1986) have examined clinical features of CMML patients in an attempt to identify prognostic factors. Although reduced survival was found to be associated with high white cell counts (Alessandrino *et al.*, 1985), overall prognostic factors have been difficult to find.

As most previous studies have examined only small numbers of patients, we examined 97 cases of CMML referred to this department for leukaemic diagnosis and assessed various blood and bone marrow features as potential prognostic factors. Additionally, as it has been suggested that MDS may be associated with an increase in second malignancies (Copplestone *et al.*, 1986; Raz *et al.*, 1984; Haznedar, 1985; Sans-Sabrafen *et al.*, 1986; Mufti *et al.*, 1983) data were collected about any other cancers present either before or after the diagnosis of CMML.

### Materials and methods

### *Patients studied*

Cases examined were referred to this department for diagnosis during the period 1981–1986. Blood and marrow specimens were taken at diagnosis into EDTA for morphological and cytochemical studies and a serum sample was also taken in most cases for lysozyme estimation. Date of birth and age at diagnosis were noted as were details of survival and the presence of second malignancies.

Correspondence: C.S. Scott.
Received 6 February 1987.

Survival data was collected by examination of case notes by one of the authors (ANS), and from the Yorkshire Regional Cancer Registry. The immediate cause of death (where applicable) was taken from the death certificate or post-mortem report and survivals calculated from initial diagnosis.

In total 97 cases were studied; 48 males and 49 females (male/female ratio 1:1) and complete survival and follow up data were available in 79 cases.

### *Morphological, cytochemical and serum lysozyme investigations*

Morphology was examined on May–Grunwald–Giemsa stained peripheral blood and bone marrow smears and the diagnosis of CMML made according to FAB criteria (Bennett *et al.*, 1982). Minimal diagnostic criteria for CMML were the presence of a total peripheral blood monocyte count of $>1\times10^9\,l^{-1}$, together with other evidence of dysplasia in the peripheral blood or bone marrow, without any clinical history to suggest a secondary cause for the monocytosis. The monocyte count was based on a standard coulter S-plus white cell plot, with a manual differential and adjusted to take account of nucleated red cells. Any evidence of dysplasia (e.g. hypogranularity of myeloid cells, presence of micromegakaryocytes, and dyserythropoiesis) was noted and features such as haemoglobin concentration, peripheral blood white cell and monocyte counts and platelet count recorded.

Cases of juvenile CGL were excluded from the study because although some features of this disease are similar to CMML, there is evidence to suggest that this condition should be considered as a separate diagnostic category (Altman *et al.*, 1974; Thomas *et al.*, 1981).

The proportions of bone marrow cells showing double (α-naphthyl acetate and chloroacetate) esterase staining, previously associated with MDS (Scott *et al.*, 1983, 1984), were cytochemically assessed by conventional techniques (Yam *et al.*, 1971).

Serum lysozyme was estimated as previously described (Milligan *et al.*, 1984) by spectrophotometric measurement of micrococcus lysodeikticus lysis; the normal range being 150–500 u ml$^{-1}$ where 1 unit is defined as the amount of enzyme causing a decrease in A450 of 0.001 min$^{-1}$ at 37°C.

### *Statistical analysis*

Individual variables were examined for survival using the log-rank test (Peto *et al.*, 1977) and also analysed separately allowing for age as a stratified variable (grouped <75 yrs and >75 yrs).

Variables found to be significant or of borderline significance at the 5% level were further investigated using Cox's proportional hazards model (Cox, 1972) both as continuous and discrete variables. Graphs of log (−log (survival function)) for one variable stratified for another were plotted to see whether there were any obvious violations of the underlying assumption of proportional hazard functions. There did not appear to be any evidence of non-proportionality.

### Results

During the period 1981–1986, 380 cases of MDS were referred for diagnosis. These included refractory anaemia (RA; $n=75$), refractory anaemia with excess sideroblasts (RAS; $n=33$), refractory anaemia with excess blasts and refractory anaemia with excess blasts in transformation (RAEB & RAEBt; $n=90$), CMML ($n=97$) and MDS unclassified ($n=85$). The latter group included patients in which insufficient data was available to confidently diagnose a particular MDS type but where a strong suspicion of MDS was present (i.e. hypogranular neutrophils and giant platelets on blood film, but only PB sample available for study). The number of cases of CMML (97/380; 25%) is higher than seen previously but it is likely that many patients with mild refractory anaemia are not referred for diagnosis, thus resulting in an apparent increase in the proportion of CMML cases. In this respect it is notable that the number of cases of RA seen ($n=75$; 20%) is lower than in other studies (Tricot *et al.*, 1985).

### *Haematological studies*

The mean peripheral blood white cell count was $20.7\times10^9\,l^{-1}$ (range $2.5$–$178.0\times10^9\,l^{-1}$) and the mean PB monocyte count $4.4\times10^9\,l^{-1}$ (range $1.0$–$45.0\times10^9\,l^{-1}$). The majority of cases had low monocyte counts (Figure 1) with only 17(17.5%) cases having a count exceeding $5.0\times10^9\,l^{-1}$. A similar distribution curve was seen for PB white count. Serum lysozyme was estimated in 59 cases with an observed range (Figure 1) of 170–15,600 u ml$^{-1}$ (mean 2,707 u ml$^{-1}$). Five cases had a serum lysozyme within the normal range.

Bone marrow monocytes ranged from 0.5%–74% (mean 12.5%) and BM double esterase positive cells from 0–24% with a mean of 4.8%. BM blasts were <10% in all cases.

Statistical relationships for the data were assessed using Spearmans non-parametric rank correlation coefficient (Table I). Absolute PB monocyte counts showed significant correlations with total WBC counts ($P<0.001$) and serum lysozyme concentrations ($P=0.005$) but, unexpectedly, there was no statistical relationship between PB monocyte counts and the proportions of BM monocytes ($P=0.5$). The proportions of DE-positive cells were however correlated with BM monocyte percentages ($P<0.01$), but not with any of the other features examined.

### *Survival data*

Survival was calculated using the Kaplan–Meier method to cancer death and this gave a median survival for all patients of 18 months from diagnosis (Figure 2). When age was taken into account (two groups <75 yrs and >75 yrs; in two cases the date of birth was unknown giving a total of 77 evaluable patients), it was found that the older age group did worse (Figure 3) although this difference was of borderline significance ($P=0.057$).

Total peripheral blood white cell counts (<10.0 versus $10.0\times10^9\,l^{-1}$ or greater) were not significant as a prognostic factor ($P=0.165$) even though there was a trend for the low count group to survive longer. Peripheral blood monocyte count (<5.0 *versus* $5.0\times10^9\,l^{-1}$ or over) was however significant ($P=0.013$) as a prognostic factor (Figure 4) with the high count group having a median survival from diagnosis of 14 months and the low count group a median survival of 2 years. Although all patients were known to have a monocyte count of $>1\times10^9\,l^{-1}$, in 7 cases the absolute number had not been documented and these were excluded from this part of the analysis.

DE positive cells in bone marrow were not a significant prognostic factor ($P=0.724$) and neither was the percentage of BM monocytes ($P=0.961$).

Serum lysozyme was divided into two groups (<2,500 and >2,500 u ml$^{-1}$); a cut-off point selected arbitrarily as five times the normal upper limit, the low lysozyme group surviving longer than those with levels exceeding 2,500 u ml$^{-1}$ ($P=0.028$; Figure 5).

**Figure 1** Peripheral blood monocyte count and serum lysozyme distributions.

**Table I** Spearman correlation coefficients for relationships between various parameters in chronic myelomonocytic leukaemia (CMML)

| | *Total WBC* ($\times10^9\,l^{-1}$) | *PB monocytes* ($\times10^9\,l^{-}$) | *BM monocytes* (%) | *BM DE +ve* (%) | *Serum lysozyme* ($u\,ml^{-1}$) |
|---|---|---|---|---|---|
| Total WBC ($\times10^9\,l^{-1}$) | — | $P<0.001$[a] | NS[b] | NS | $P=0.002$ |
| PB monocytes ($\times10^9\,l^{-1}$) | $P<0.001$ | — | NS | NS | $P=0.003$ |
| BM monocytes (%) | NS | NS | — | $P=0.007$ | NS |
| BM DE +ve cells (%) | NS | NS | $P=0.007$ | — | NS |
| Serum lysozyme (u ml$^{-1}$) | $P=0.002$ | $P=0.003$ | NS | NS | — |

[a]Results expressed as $P$ values for any given Spearman coefficient value; [b]NS = not significant.

**Figure 2** Survival in CMML – All cases ($n=79$).

**Figure 3** Survival in CMML by age group ($n=77$).

**Figure 4** Survival in CMML by blood monocyte count ($n=72$).

**Figure 5** Survival in CMML by serum lysozyme level ($n=59$).

*Multiple regression analysis*

Cox's proportional hazards regression model was used to investigate the prognostic importance of the variables in a continuous format (Table II). With various combinations, only serum lysozyme was consistently selected by the automatic stepwise selection procedure. In discrete format, serum lysozyme was again consistently selected, but PB monocytes and age also appeared to provide additional prognostic information. It was concluded from this analysis that serum lysozyme was the most important prognostic factor of those examined, both in continuous and discrete format. Blood monocytes and age also affected prognosis but were more noticeable in their effect when grouped into discrete variables.

**Table II** Cox's model analysis for variables in continuous format

| *Variables investigated* | *Variables selected (P value)* |
|---|---|
| Serum lysozyme, PB monocytes | Serum lysozyme ($P=0.002$) |
| Serum lysozyme, age | Serum lysozyme ($P=0.003$) |
| PB monocytes, age | Neither entered |
| Serum lysozyme, age, PB monocytes | Serum lysozyme ($P=0.002$) |

*Second malignancies*

In the 79 patients where accurate survival and follow-up were available we found 10 cases of carcinoma (2 skin, 2 colonic, 1 breast, 1 ovarian, 1 prostate, 1 bladder and 1 uterus) with one patient having metastatic brain deposits with unknown primary. In addition two CMML patients had myeloma and one had symptomless paraproteinaemia with a monoclonal increase in BM plasma cells (15%). In both myeloma patients, CMML and myeloma were diagnosed simultaneously but in the carcinoma group 7/10 were diagnosed as having carcinoma before the diagnosis of CMML was made. Using the 'Man Years' technique (for comparing observed with expected incidences of diseases in age and sex matched groups), it was found that no statistical increase in cancers was present either for the CMML group as a whole (O/E ratio 1.21, $P=0.297$) or for male (O/E ratio 0.84, $P=0.708$) or female (O/E ratio 1.75, $P=0.11$) groups individually.

Four patients developed acute myeloid leukaemia (1 FAB M2, 3 FAB M4) defined as >30% of nucleated cells, excluding erythroid precursors, being blasts, subsequent to the diagnosis of CMML. One further patient presented with AML (FAB M2), who following induction chemotherapy, remitted with a typical picture of CMML which was maintained for 14 months before relapsing. None of the patients with AML were considered as having true second malignancies since it is likely that in these cases the AML develops from the same clone as the CMML, and can reasonably be regarded as a progression of the original disease.

### Discussion

CMML is a relatively uncommon disease predominantly of the elderly for which, in the majority of cases, aggressive therapeutic regimens are inappropriate. However, if it were possible to identify patients who had a poor prognosis then, apart from the obvious benefits of having prognostic information available on any given patient, treatment might be more rationally planned.

We have shown that CMML patients with a high monocyte count do worse than those with lower PB monocyte counts but this did not appear to be related to monocytic tumour load, as reflected by monocytic infiltration of bone marrow. In contrast to a recent study (Groupe Francais de Cytogenetique, 1986), which reported 59 cases of high count ($>5.0\times10^{9}\,l^{-1}$) CMML in a total of 120 (49.2%) patients, we found only a small number (17/97: 17.5%). Reasons for this discrepancy are unclear but may be related to sampling methods and diagnostic criteria employed. In our survey, all cases with primary dysplasia referred from a wide population area were examined as potential 'low count' CMML and, conversely, we reviewed all cases of atypical chronic granulocytic leukaemia (CGL) and myeloproliferative disease as potential 'high count' cases, and cases were diagnosed as CMML only if they fulfilled the FAB criteria for this diagnosis. It is also accepted that it can be difficult to differentiate between $Ph^1$ negative CGL and CMML.

Increased BM monocytic components were found in many of our cases (mean 12.5% BM monocytes), in contrast to early morphological descriptions (Broun, 1969; Miescher & Farquet, 1974), but were not significantly correlated with absolute PB monocyte counts. An increase in BM monocytes was however associated with the presence of abnormal numbers of double esterase (DE)-positive cells. It has been shown previously that DE +ve cells in the BM in MDS are probably of granulocytic origin (Scott *et al.*, 1984) so the association of DE +ve cells and BM monocytes cannot be explained on the basis that the same cells are being stained. Rather it would appear that the increased monocytic involvement of BM is associated with the abnormality (possibly due to gene derepression) in enzyme expression that causes granulocyte precursors to express monocyte-associated ANA in addition to chloroacetate esterase.

As in leukaemia generally, age significantly influenced prognosis, independently of the other factors examined. However, it is difficult to say whether this means the disease is inherently more aggressive in an older population or whether, as might be expected, older people simply have a higher overall mortality with the CMML contributing in a non-specific way.

Serum lysozyme was the single most valuable prognostic factor whether taken as a discrete or continuous variable. Serum lysozyme is elevated in many types of myeloid leukaemias, particularly of monocytic type (Milligan *et al.*, 1984; Norfolk *et al.*, 1985; Scott *et al.*, 1985), and shows some correlation with serum beta-2 microglobulin levels in monocytic proliferations (Norfolk *et al.*, 1985). The concentrations of these serum components are considered to reflect the degree of monocytic infiltration, and hence tumour load in CMML, and this supports our observation that serum lysozyme is a strong prognostic indicator. However, it is of interest that serum lysozyme was not related to BM monocyte count, which also might be thought to provide evidence of monocytic tumour mass. One possible explanation for this is that the BM monocyte count may be a poor indicator of monocytic load in CMML, as is the bone marrow blast cell count in other types of leukaemia, and that serum lysozyme more accurately reflects total body monocytic tumour load and turnover analagous to LDH enzymes in malignancies (haemopoietic and non-haemopoietic) generally (Ho *et al.*, 1982; Stefani, 1985; Scott *et al.*, 1986). Five cases had a normal serum lysozyme but fulfilled the FAB criteria for the diagnosis of CMML in every other way, and 4 of these had >10% BM monocytes. The occurrence of a normal serum lysozyme in occasional patients with CMML is therefore unusual, but not without precedence as cases of well differentiated acute monocytic leukaemia (M5b) may also have a normal serum lysozyme (Norfolk *et al.*, 1985).

The median survival in our study (18 months) was similar to two earlier studies (Solal–Celigny *et al.*, 1984; Alessandrino *et al.*, 1985; median survivals 15.8 and 18 months respectively), although less than that reported recently (Groupe Francais de Cytogenetique Hematologique, 1986; median survival 27 months), confirming that the disease has a poor prognosis with few patients surviving more than two years following clinical presentation.

It has also been suggested that the incidence of second malignancies in CMML may be increased (Copplestone *et al.*, 1986; Mufti *et al.*, 1983) and some authors consider that a picture of CMML may occur as part of a paraneoplastic syndrome (Haznedar, 1985; Raz *et al.*, 1984; Sans-Sabrafen *et al.*, 1986). Seven of our series of patients had a carcinoma present before the diagnosis of CMML was made and it is therefore possible that in these cases the picture of dysplasia and monocytosis was a result of the carcinoma, rather than representing the development of a new tumour. However, when the 10 cases of carcinoma and 2 of myeloma were analyzed against an age and sex matched group, no statistical increase of second malignancy was found.

It can be argued that since paraprotein was not sought routinely in all cases it is possible that the true incidence of myeloma was underestimated. While this may be true, increased plasma cells were only found in one bone marrow (apart from those with myeloma) and none of the other patients had any of the clinical or biochemical features of the disease. Paraprotein in CMML has been found in the absence of increased BM plasma cells (Barnard *et al.*, 1979) but this is presumably uncommon as all 12 patients with paraproteinaemia in a recent study (Group Francais de Cytogenetique Hematologique, 1986) showed some increase in BM plasma cells. It has been proposed in another study (Copplestone *et al.*, 1986) that the occurrence of B-cell malignancy in MDS does not represent a true second tumour and may be part of the same clonal disturbance, though we found no direct evidence for this in our study.

In conclusion, we have analysed 97 cases of CMML for prognostic factors and found that age, PB monocyte count and serum lysozyme levels are significant single independent prognostic factors. Multiple regression analysis showed serum lysozyme to be the most important of these although the other factors remained significant even when lysozyme was taken into account. Use of these easily available features should allow better prediction of survival in CMML.

We thank Drs A. Antonis, I.C. Balfour, D.L. Barnard, J.A. Child, A.T. Edwards, M.S. Edwards, M.C. Galvin, K. Hunt, M.M. McEvoy, S. Mayne, R.D. Pyrah, L.A. Parapia, R. Sibbald and J.G. Tetley for allowing us to study their patients.

We especially thank the Yorkshire Regional Cancer Registry for providing much of the data and Dr R.A. Cartwright and staff of the Yorkshire Regional Cancer Organisation (Epidemiology) for their help in interpretation of data.

Work in this Department is supported by the LRF and the Friends of the Leukaemia Unit, Leeds General Infirmary, and many of the cases examined were submitted as part of the LRF Data Collection (Epidemiology) Study. ANS is an LRF Training Fellow.

### References

ALESSANDRINO, E.P., ORLANDI, E., BRUSAMOLINO, M. & 4 others (1985). Chronic myelomonocytic leukaemia: Clinical features, cytogenetics, and prognosis in 30 consecutive cases. *Haematol. Oncol.*, **3**, 147.

ALTMAN, A.J., PALMER, C.G. & BAEHNER, R.L. (1974). Juvenile 'chronic granulocytic' leukaemia: A panmyelopathy with prominent monocytic involvement and circulating monocyte colony-forming cells. *Blood*, **43**, 341.

BARNARD, D.L., BURNS, G.F., GORDON, J. & 4 others (1979). Chronic myelomonocytic leukaemia with paraproteinaemia but no detectable plasmacytosis. *Cancer*, **44**, 927.

BENNETT, J.M., CATOVSKY, D., DANIEL, M.-T. & 4 others (1982). Proposals for the classification of the myelodysplastic syndromes. *Br. J. Haematol.*, **51**, 189.

BROUN, G.O. (1969). Chronic erythromonocytic leukaemia. *Am. J. Med.*, **47**, 785.

COPPLESTONE, J.A., MUFTI, G.J., HAMBLIN, T.J. & OSCIER, D.G. (1986). Immunological abnormalities in myelodysplastic syndromes (II). *Br. J. Haematol.*, **63**, 149.

COX, D.R. (1982). Regression models and life tables (with discussion). *J. R. Statist. Soc.*, B. **34**, 187.

GEARY, C.G., CATOVSKY, D., WILTSHAW, E. & 7 others (1975). Chronic myelomonocytic leukaemia. *Br. J. Haematol.*, **30**, 289.

GROUPE FRANCAIS DE CYTOGENETIQUE (1986). Cytogenetics of chronic myelomonocytic leukaemia. *Cancer Genet. Cytogenet.*, **21**, 11.

HAZNEDAR, R. (1985). Pancytopaenia with a hypercellular bone marrow as a possible paraneoplastic syndrome. *Am. J. Haematol.*, **19**, 205.

HO, A.D., FIEHN, W. & HUNSTEIN, W. (1982). Intracellular lactic dehydrogenase and phosphohexose isomerase activity in leukaemia and malignant lymphoma. *Br. J. Haematol.*, **50**, 637.

LINMAN, J.W. (1970). Myelomonocytic leukaemia and its preleukaemic phase. *J. Chron. Dis.*, **22**, 713.

MIESCHER, P.A. & FARQUET, J.J. (1974). Chronic myelomonocytic leukaemia in adults. *Sem. Haematol.*, **11**, 2, 129.

MILLIGAN, D.W., ROBERTS, B.E., LIMBERT, H.J., JALIHAL, S. & SCOTT, C.S. (1984). Cytochemical and immunological characteristics of acute monocytic leukaemia. *Br. J. Haematol.*, **58**, 391.

MILNER, G.R., TESTOR, G.M., GEARY C.G. and 4 others (1977). Bone marrow culture studies in refractory cytopenias and 'smouldering leukaemia'. *Br. J. Haematol.*, **35**, 251.

MUFTI, G.J., HAMBLIN, T.J., CLEIN, G.P. & RACE, C. (1983). Coexistent myelodysplasia and plasma cell neoplasia. *Br. J. Haematol.*, **54**, 91.

NORFOLK, D.R., DAVY, M., FORBES, M.A. & SCOTT, C.S. (1985). Serum B-2-microglobulin and lysozyme concentrations in 101 cases of untreated acute myeloid leukaemia. *Disease Markers*, **3**, 177.

PETO, R., PIKE, M.C., ARMITAGE, P. & 7 others (1977). Design and analysis of randomized clinical trials requiring prolonged observation of each patient II. Analysis and examples. *Br. J. Cancer* **35**, 1.

RAZ, I., SHINAR, E. & POLLIACK, A. (1984). Pancytopaenia with hypercellular bone marrow – A possible paraneoplastic syndrome in carcinoma of the lung: A report of three cases. *Am. J. Haematol.*, **16**, 403.

SAARNI, M. & LINMAN, J.W. (1971). Myelomonocytic leukaemia: Disorderly proliferation of all marrow cells. *Cancer* **27**, 5, 1221.

SANS-SABRAFEN, J., WOESSNER, S., BESSES, C., LAFUENTE, R., FLORENSA, L. & BUXO, J. (1986). Association of chronic myelomonocytic leukaemia and carcinoma: A possible paraneoplastic myelodysplasia (letter). *Am. J. Haematol.*, **22**, 109.

SEXAUER, J., KASS, L. & SCHNITZER, B. (1974). Subacute myelomonocytic leukaemia. Clinical, morphologic, and ultrastructural studies of 10 cases. *Am. J. Med.*, **57**, 853.

SCOTT, C.S., CAHILL, A., MORGAN, M. & ROBERTS, B.E. (1984). Double esterase positive cells (letter). *Br. J. Haematol.*, **58**, 762.

SCOTT, C.S., CAHILL, A., BYNOE, A.G., AINLEY, M.J., HOUGH, D. & ROBERTS, B.E. (1983). Esterase cytochemistry in primary myelodysplastic syndromes and megaloblastic anaemias: Demonstration of abnormal staining patterns associated with dysmyelopoiesis. *Br. J. Haematol.*, **55**, 411.

SCOTT, C.S., MORGAN, M.A.M., LIMBERT, H.J., MACKARILL, I.D. & ROBERTS, B.E. (1985). Cytochemical, immunological and ANAE-isoenzyme studies in acute myelomonocytic leukaemia: A study of 39 cases. *Scand. J. Haematol.*, **35**, 284.

SCOTT, C.S., DAVEY, M., HAMILTON, A. & NORFOLK, D.R. (1986). Serum enzyme concentrations in untreated acute myeloid leukaemia. *Blut*, **52**, 297.

SOLAL-CELIGNY, P., DESAINT, B., HERRERA, A. & 8 others (1984). Chronic myelomonocytic leukaemia according to FAB classification: Analysis of 35 cases. *Blood*, **63**, 634.

STEFANI, M. (1985). Enzymes, isoenzymes, and enzyme variants in the diagnoses of cancer. *Cancer*, **55**, 1931.

THOMAS, W.J., NORTH, T.B., POPLACK, D.G., SLEASE R.B. & DUVAL-ARNOULD, B. (1981). Chronic myelomonocytic leukaemia in childhood. *Am. J. Haematol.*, **10**, 181.

TRICOT, G., VLIENTINCK, R., BOOGAERTS, B. & 4 others (1985). Prognostic factors in the myelodysplastic syndromes: Importance of initial data on peripheral blood counts, bone marrow cytology, trephine biopsy and chromosomal analysis. *Br. J. Haematol.*, **60**, 19.

YAM, L.T., LI, C.Y. & CROSBY, W.H. (1971). Cytochemical identification of monocytes and granulocytes. *Am. J. Clin. Pathol.*, **55**, 283.